# Beyond the screen: enhancing ethnic cultural representation and engagement through immersive 360° documentary experiences in museums

**DOI:** 10.1057/s41599-025-05429-z

**Published:** 2025-07-04

**Authors:** Xiaolin Sun, Eugene Ch’ng

**Affiliations:** 1 University of Nottingham Ningbo China, Ningbo, China; 2 Beijing Normal-Hong Kong Baptist University, Zhuhai, China

**Keywords:** Cultural and media studies, Anthropology, Cultural and media studies

## Abstract

In this era of digital heritage, immersive media, such as 360° documentaries, have been proposed to enrich engagement and learning. However, how immersive 360° media formats influence the understanding of ethnic minority cultures remains underexplored. We conducted a controlled laboratory experiment comparing traditional 2D and 360° documentary presentations of the Miao community in Guizhou Province, using the same narration and content, including village scenes, performances, and crafts, to isolate the effect of media format. The results suggest that the 360° documentary may increase viewers’ enjoyment, sense of presence, curiosity, and appreciation of cultural content relative to a comparable 2D film. These exploratory findings indicate a potential for immersive media to deepen cultural understanding. Our study underscores 360° documentaries as a promising tool for engaging audiences with ethnic minority cultures, while noting that these conclusions are provisional given the lab setting and should be tested in real-world museum contexts.

## Introduction

Rapid advances in digital technologies have transformed the presentation of cultural heritage, with immersive 360° documentaries drawing growing interest for their potential to enhance audience engagement (Nagakura et al., 2017; Škola et al., 2020). These media offer viewers a panoramic and interactive view of environments, which can support richer narrative immersion. Despite their increasing adoption, the specific role of 360° documentaries in communicating ethnic minority heritage remains underexplored. This question is particularly relevant in contexts such as Chinese ethnic exhibitions, which aim to reflect the country’s “unity in diversity” (Leung, 2023) but often rely on static displays and informational digital screens (Sun & Ch’ng, 2024). Visitor feedback from the Guizhou Provincial Museum, for instance, has revealed strong interest in more immersive and interactive digital experiences to convey deeper cultural meaning (Sun & Ch’ng, 2024).

In this context, our study investigates whether immersive 360° documentaries offer advantages over traditional 2D formats in fostering engagement with cultural content. We conducted a controlled laboratory experiment comparing two versions of the same short documentary about Miao village, presented either in 2D on a screen or in 360° via a head-mounted display. Our aim was to isolate the effect of media format on four key engagement dimensions, namely enjoyment, presence, curiosity, and appreciation. These categories are well-established in engagement theory (Csikszentmihalyi, 2008; Ryan & Deci, 2000; Witmer & Singer, 1998) and are relevant to both museum experiences and digital storytelling. The laboratory setting allowed us to minimise contextual variables typically found in museums, such as exhibition layout, ambient noise, distractions from visitors, and group behaviour. While this design limits ecological validity, it provides a necessary first step in understanding the impact of media format under controlled conditions. Subsequent research will be needed to explore how such findings apply in actual museum settings.

Our approach is inspired by a broader shift in museum practice from object-centred to visitor-centred engagement (Dierking & Falk, 1998; Hein, 2000). Museums worldwide are increasingly incorporating interactive technologies to foster more meaningful connections between visitors and exhibits. Extended reality (XR) is one promising avenue for this transformation. Song and Evans (2024) note that multisensory XR technologies can heighten presence, enabling audiences to appreciate the “narrative dimension” of cultural artefacts more fully. Related scholarship on technological embodiment suggests that immersive experiences can foster a progressive integration between user and medium, through stages of exploration, adaptation, and eventual symbiosis (Ahmedien, 2024). We assume that 360° documentaries may encourage this kind of embodied engagement by surrounding viewers with a cultural environment in ways not possible in traditional formats.

By comparing 2D and 360° formats under same conditions, this exploratory study offers preliminary insights into how immersive media might influence cultural engagement. We combine quantitative survey results with qualitative feedback to assess the immediate effects on viewer experience. As a lab-based investigation, the findings are necessarily tentative and should be interpreted as an initial step toward understanding the role of immersive formats in cultural communication. The following sections outline relevant literature on immersive media and digital exhibitions, describe our methodology, present the results, and discuss their implications and limitations.

## Literature review

Museums have increasingly evolved from static, object-centric institutions into dynamic, visitor-centred spaces (Samis & Michaelson, 2016). This shift is reflected in the transition from early anthropological display models to approaches that foreground visitor experience. Falk and Dierking’s Interactive Experience Model frames museum visits as shaped by overlapping personal, social, and physical contexts (Falk & Dierking, 2018). Simon (2010) further proposes that museums should not treat visitors as passive observers but engage them as active cultural participants. These frameworks collectively emphasise the importance of multisensory, personalised, and interactive encounters in cultural engagement.

This conceptual evolution aligns with technological developments that support participatory and immersive experiences. Extended reality (XR), encompassing 360° media, AR, and VR, has become central to these efforts. Song and Evans (2024) argue that multisensory XR can redefine museum interactions by creating “thingly” encounters, in which visitors form embodied relationships with artefacts. This is echoed in Zhang et al., (2024) bibliometric analysis, which highlights growing interest in the use of immersive technologies such as VR and AR in Chinese museums to enhance engagement and cultural representation. The growing application of immersive technologies reflects a broader move from information delivery to experiential learning, offering audiences agency, emotion, and sensory richness in how they connect with cultural material.

In psychological terms, immersive engagement is supported by Flow Theory and Self-Determination Theory. Csikszentmihalyi (2008) describes flow as a mental state of deep absorption, often triggered when an activity balances challenge and skill. Ryan and Deci’s (2000) Self-Determination Theory identifies autonomy, competence, and relatedness as key drivers of intrinsic motivation, which are frequently cited in educational and museum contexts. These dimensions are increasingly relevant in immersive media, where freedom to explore and interpret cultural content can foster emotional and cognitive investment. Presence, defined as “the extent to which the unification of simulated sensory data and perceptual processing produces a coherent ‘place’ that you are ‘in’”(Slater, 2003), plays a particularly important role in shaping engagement. For instance, Choi and Nam (2024) found that virtual reality experiences offering existential authenticity significantly enhanced visitor satisfaction and motivation to revisit. Similarly, Liu and Sutunyarak (2024) observed that presence and embodiment in VR positively influenced behavioural intentions to engage with cultural heritage.

These theoretical models are reflected in international museum practices. The Science Museum in London launched a VR exhibit simulating astronaut Tim Peake’s descent from space, allowing visitors to virtually experience re-entry aboard the Soyuz capsule (Jones, 2021). The Smithsonian Renwick Gallery developed “No Spectators: The Art of Burning Man,” a VR project that offers virtual tours of large-scale participatory artworks from the Burning Man festival, recreated digitally and accessible through a social VR platform (Li et al., 2023). In Southeast Asia, the National Museum of Singapore’s “Story of the Forest” uses dynamic panoramic imaging to immerse visitors in a digital forest, drawing attention to local biodiversity through an experiential lens (Dong, 2024). In China, Peking University’s Sackler Museum recreated the spatial context of 11th Century Buddhist murals through VR, allowing visitors to explore a reconstructed temple hall in which the artefacts originally appeared (Zhang & Zuo, 2020). These cases illustrate how immersive media extend narrative depth, presence, and access, while offering new ways to spatially and emotionally situate cultural material.

Despite these advances, the application of immersive technologies to represent ethnic minority cultures, particularly in China, remains underdeveloped. Many Chinese museums still rely on traditional display strategies. Ethnic costumes are often exhibited on mannequins or flat platforms behind glass, while accessories and textiles are mounted as visual specimens (Varutti, 2014). Though some institutions incorporate digital enhancements, these are typically limited to informational screens or basic interactives. For intangible cultural heritage, such as embroidery or weaving, tools like looms or dye vats may be displayed, but without demonstrating the processes or situating them in lived cultural contexts. This static presentation style limits narrative depth and emotional engagement.

Recent empirical work, however, suggests growing audience interest in immersive alternatives. At the Guizhou Provincial Museum, AR applications have begun to appear alongside traditional displays (Sun & Ch’ng, 2024). Sun and Ch’ng (2024) also report that museum visitors expressed strong interest in further immersive technologies, particularly to contextualize artefacts within everyday cultural practices. This aligns with broader calls to use XR in preserving and disseminating intangible cultural heritage (ICH). Scholars such as Oladokun et al. (2024) and Innocente et al. (2024) emphasise that digital environments can preserve performance, ritual, and oral tradition in ways not possible through static documentation alone.

Theoretical work on technological embodiment supports this perspective. Ahmedien (2024) describes a process of human–technology symbiosis in which users move through stages of exploration, adaptation, and embodiment. This process allows immersive media to reshape perception and interaction by gradually integrating users into the medium. Such perspectives are helpful when evaluating 360° documentaries, which, although less interactive than full VR, still enable viewers to look freely within a filmed environment and engage spatially with cultural content. Studies comparing 2D and 360° formats indicate that 360° can enhance both affective responses and cognitive curiosity, particularly in educational settings (Ausin-Azofra et al., 2021; Lampropoulos et al., 2021; Škola et al., 2020).

In sum, the integration of immersive media into museum practice reflects both theoretical and empirical insights about engagement, presence, and learning. While several global institutions have demonstrated XR’s potential, there is a noticeable gap in applying these methods to the representation of ethnic minority cultures. This study aims to address that gap by evaluating whether a 360° documentary featuring Miao cultural scenes can improve engagement metrics relative to a traditional 2D version. By combining theories of flow, presence, and embodiment with museum-based motivations, the research contributes to ongoing discussions about the role of immersive technologies in cultural representation and audience experience.

## Materials and methods

### Materials

This study used two documentary formats, a traditional 2D version and an immersive 360° version, both presenting the same narration and content focused on the Miao communities in less-touristed areas of the Qiandongnan Miao and Dong Autonomous Prefecture. The 2D version showed a fixed viewpoint, whereas the 360° version allowed viewers to explore the environment from all angles. Both documentaries depict village settings, performances, and traditional craftsmanship of the Miao community. The Miao community was selected due to its distinctive cultural beliefs, festivals, and craftsmanship, which symbolise the rich diversity of ethnic minority cultures in Chinese museums (Varutti, 2014). Filming in less-touristed locations aimed to preserve authenticity and cultural integrity.

Each documentary ran for 7 min and 5 s, ensuring that any differences in viewer engagement could be attributed to the format rather than the content. Table 1 provides an overview of the documentary scenes.

**Table 1:** Documentaries overview.

No	2D View	360° View Equirectangular Panorama	Description
1			The documentary begins with the renowned Miao architectural form, the lounge bridge.
2			The shot shows the third step of batik-making—boiling. A practitioner boils dyed textiles in boiling water to dewax.
3			The shot captures an elderly Miao woman embroidering skillfully.
4			The documentary ends with a scene of the Miao people performing the Lusheng Dance in a plaza.

## Methods

### Design

This study employed a between-subjects experimental design to evaluate participants’ engagement with documentaries about Miao culture presented in traditional 2D and immersive 360° formats. Participants were randomly assigned to one of these formats, allowing a direct comparison of how each format influences key engagement factors, including enjoyment, presence, curiosity, and cultural appreciation. We also recorded participants’ prior familiarity with Miao communities to explore whether existing cultural exposure affected their responses.

In line with Csikszentmihalyi’s (2008) Flow Theory, the documentaries were designed to balance cognitive challenge with participants’ comfort level, aiming to induce a state of flow and thereby enhance engagement. The questionnaire design was informed by Ryan and Deci’s (2000) Self-Determination Theory, emphasising autonomy, competence, and relatedness to deepen motivation and enrich learning.

All experiments were conducted in a controlled laboratory setting to ensure internal validity and isolate the effect of the media format from external variables, such as visitor flow and social interactions. This approach provides a clear foundation for comparing the impacts of the 2D and 360° formats. However, we recognise that real museum contexts introduce additional factors that can influence engagement. Future research should test these formats in actual museum environments to validate and extend our findings in dynamic, real-world contexts.

### Materials preparation and equipment setup

In-depth consultation with Miao community representatives preceded filming to ensure an authentic portrayal of cultural traditions and daily activities. This process enabled access to community events and homes, allowing us to film intimate aspects of daily life. The representatives advised excluding the silver jewellery production process from the documentary due to its complexity, despite its importance to Miao costume culture.

An Insta360 X3 camera captured over 500 GB of 360° video footage of village settings, dance performances, and craftsmanship. In post-production, the most representative clips were selected and refined using Insta360 Studio, Adobe Premiere Pro 2024, After Effects 2024, and Photoshop 2024. Key enhancements included removing shadows from tripods and selfie sticks. Narration was generated using WellSaid Labs’ AI voice synthesis, employing a clear and engaging voice avatar. Several Miao community members reviewed the final documentary for authenticity and cultural accuracy before exporting it in both formats for the experiment.

**Fig. 1:**
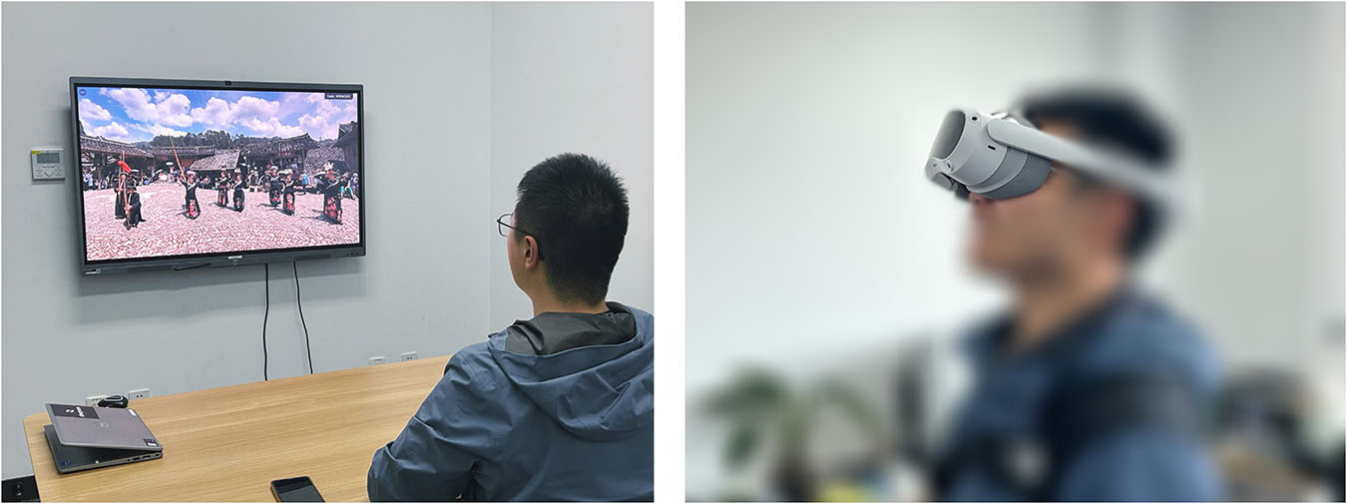
Participant engagement with the 2D and 360° documentaries. This figure shows participants during the screenings of the 2D and 360° documentaries in a controlled laboratory setting. It illustrates the different ways viewers interacted with the two formats, highlighting the contrast between traditional screen-based viewing and immersive VR experiences.

During the experiment, participants in the 2D condition viewed the documentary on a 55-inch MAXHUB TV, while those in the 360° condition viewed it using a Pico 4 VR headset. Participants received guidance on how to use the VR equipment comfortably. Figure 1 illustrates participants engaging with each documentary format.

### Participants

Participants were recruited via stratified random sampling and snowball sampling, targeting approximately 80 individuals evenly distributed across the two documentary formats and stratified by prior exposure to Miao communities. Recruitment began through WeChat; however, due to a lower-than-expected response from individuals who had visited Miao communities, we supplemented with snowball sampling to increase participation. From 138 applicants, 94 participants (52 female, 42 male; aged 16–45) were selected based on self-reported English proficiency, which was necessary to understand the documentary narration.

Most respondents were younger individuals, a trend consistent with earlier fieldwork at the Guizhou Provincial Museum, where existing digital exhibits primarily attract a digitally proficient younger audience (Sun & Ch’ng, 2024). As a result, our final sample skewed toward this demographic rather than representing all age groups. Future studies should include a broader age range and varying levels of digital familiarity to provide a more comprehensive view of how immersive technologies influence diverse populations.

### Procedure

Informed consent was obtained from all participants, who were briefed on the study’s purpose and procedures and assured of their right to withdraw at any time. Participants first completed a pre-viewing questionnaire collecting demographic information, self-reported curiosity about Miao communities and culture, and viewing habits. Based on self-reported medical conditions (e.g., vertigo or claustrophobia) and comfort with VR, participants were then assigned to view either the 2D or 360° documentary.

After viewing the assigned documentary, participants completed a post-viewing questionnaire measuring enjoyment, sense of presence, changes in curiosity, and appreciation of cultural content. An open-ended question invited them to describe any new insights or changes in perception about Miao culture. All data were anonymized and securely stored to protect participant privacy. We excluded 10 of the initial 94 participants due to unusually short completion times or inconsistent responses about prior visits to Miao communities, leaving 84 participants (42 per format) in the final analysis. These groups were balanced with respect to prior Miao community experience.

### Measure

Participants responded to a series of statements using seven-point Likert scales, ranging from 1 (strongly disagree) to 7 (strongly agree). The questionnaires focused on four key engagement factors, including enjoyment, presence, curiosity, and cultural appreciation. Enjoyment was measured directly by asking participants how much they enjoyed the documentary, which serves as a critical indicator of whether the content successfully captured their interest and sustained attention.

The sense of presence was evaluated using adapted scales from Witmer and Singer (1998), Qin, Rau, and Salvendy (2009), and Szita et al. (2024). Statements such as *“I was so engrossed in the documentary that I lost track of time”* and *“The documentary’s details and portrayal made me feel immersed, as if I were personally experiencing the Miao culture”* assessed the degree to which participants felt mentally and emotionally present in the documentary environment. This factor is particularly relevant in exploring how immersive formats support cultural engagement.

Curiosity was measured both before and after viewing to capture any changes in interest stimulated by the documentaries. Before watching, participants indicated their level of interest in visiting Miao communities in the future. Following the viewing, they responded to statements such as *“The documentary sparked my interest in visiting Miao communities in the future”* to assess whether the content successfully enhanced their curiosity. Comparing pre- and post-viewing responses allowed us to determine the documentaries’ effectiveness in fostering a desire for further exploration of Miao culture.

Cultural appreciation was assessed through participants’ reflections on the artistic and cultural significance of the content. Statements such as *“The documentary heightened my appreciation for the artistry and aesthetic value of the Miao people’s traditional textiles and costumes”* and *“The documentary emphasised the significance of preserving the Miao people’s intangible cultural heritage, making me value its preservation more”* captured how effectively the documentaries deepened participants’ valuation of ethnic minority cultures.

To complement these quantitative measures, the post-viewing questionnaire also included an open-ended question inviting participants to describe how their perception of Miao culture had changed after watching the documentary and to share any new insights. This qualitative feedback provided richer perspectives on the documentaries’ impact, revealing personal reflections and nuanced shifts in understanding that standard survey items may not fully capture.

### Note on laboratory vs. museum settings

We used a controlled laboratory setting to compare the effects of the 2D and 360° formats under consistent conditions. This setup isolated the influence of media format from other factors present in a museum, such as group dynamics, exhibit layout, and ambient distractions (Falk & Dierking, 2018). In both the 2D and 360° experiences, the viewer’s gaze was guided by the documentary narrative, a factor independent of the setting. By isolating the formats in this initial study, we provide a foundational understanding of their effects. Future research should test these formats in actual museum environments to validate and extend our findings, offering deeper insight into how immersive technologies like 360° documentaries enhance museum experiences.

## Results

### Descriptive data

The questionnaires explored multiple aspects of the viewing experience to assess how different documentary formats influenced engagement. These aspects included enjoyment, sense of presence, curiosity, and appreciation of cultural content, with the descriptive statistics summarised in Table 2.

**Table 2:** Descriptive statistics.

Combined Variable	Variables	2D		360°	
		x̄	σ	x̄	σ
**Enjoyment**	How much did you enjoy the documentary?	5.119	0.993	5.786	0.951
**Sense of Presence**	I was so engrossed in the documentary that I lost track of time.	4.048	1.667	5.429	1.213
	I felt an emotional connection with the subjects of the documentary, as if I was there alongside them.	3.714	1.551	5.238	1.284
	I felt I was in locations and scenarios depicted, and the cultural rituals and traditions made me feel like an active participant rather than just a viewer.	3.381	1.464	5.357	1.559
	The documentary’s details and portrayal make me feel immersed and personally experiencing the Miao culture.	3.690	1.473	5.857	1.002
**Curiosity**	(Pre-viewing) I have an interest in visiting Miao communities in the future.	4.881	1.435	4.929	1.520
	(Pre-viewing) How curious are you about specific aspects of Miao culture, for example, Miao’s history, folk dance, and traditional craftsmanship?	4.762	1.650	4.738	1.270
	(Post-viewing) The documentary sparked my interest in visiting Miao communities in the future.	4.810	1.311	5.429	1.085
	(Post-viewing) The documentary inspired my curiosity about specific aspects of Miao cultures, such as Miao’s history, folk dance and traditional craftmanship.	5.071	1.404	5.429	1.151
**Appreciation**	The documentary heightened my appreciation for the artistry and aesthetic value of the Miao’s traditional textiles and costumes.	5.071	1.276	5.262	0.989
	The documentary heightened my respect for the intricacies and skills involved in the Miao’s traditional craftsmanship.	5.476	1.254	5.405	1.231
	After watching the batik section, I respect these practitioners for their persistence in doing one thing throughout their lives.	5.619	1.306	5.905	1.226
	The documentary emphasised the significance and need for preserving the Miao’s intangible cultural heritage, making me value its preservation more.	5.214	1.507	5.452	1.435

### Data findings and visualisations

This study evaluated viewer engagement among 84 participants (45 females and 39 males), aged between 16 and 45 years, who viewed either a traditional 2D or an immersive 360° documentary. Each format was presented to 42 participants, ensuring a balanced distribution of individuals with and without prior exposure to Miao culture. This design enabled a focused analysis of how documentary format influenced engagement with Miao cultural content.

**Fig. 2:**
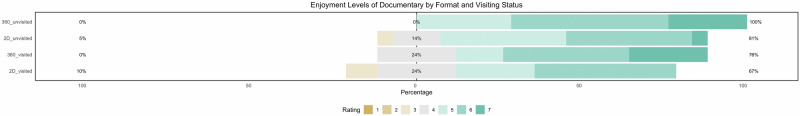
Enjoyment levels by documentary format and prior visitation. This figure presents box plots comparing participants’ enjoyment levels based on their prior visitation to the Miao community and the documentary format viewed (2D or 360°). Enjoyment was rated on a Likert scale from 1 (Did Not Enjoy at All) to 7 (Extremely Enjoyed), with ratings of 5–7 considered positive, 4 neutral, and 1–3 negative. The figure was generated using R Studio.

Figure 2 illustrates the enjoyment levels of participants grouped by documentary format (2D or 360°) and prior visitation to Miao communities. Among those without prior exposure, all participants who viewed the 360° format reported high enjoyment (ratings of 5 to 7 on the Likert scale), while 81% (*n* = 17) of those viewing the 2D format provided positive ratings; the remaining offered neutral (*n* = 3) or negative feedback (*n* = 1). Among participants who had previously visited Miao communities, 76% (*n* = 16) of those in the 360° condition gave positive ratings, compared to 67% (*n* = 14) in the 2D group, with the remainder offering neutral (*n* = 5) or negative (*n* = 2) evaluations.

Statistical analysis using the Mann-Whitney U test, which is appropriate for non-parametric ordinal data such as Likert-scale responses, supports these observations. For participants without prior exposure to Miao communities, a significant difference in enjoyment was observed between the two formats (*U* = 130.5, *p* < 0.05). In contrast, among participants who had previously visited Miao communities, no statistically significant difference in enjoyment was found between the formats (*U* = 153, p = 0.078). We also examined whether prior visitation significantly influenced enjoyment across both formats. The results indicate no significant effect, as the p-values obtained from the Mann-Whitney U test were not significant for either format (2D: *U* = 197.5, *p* = 0.550; 360°: *U* = 189.5, *p* = 0.418). These findings suggest that while the immersive 360° format is particularly effective in enhancing enjoyment for participants unfamiliar with the culture, the impact of direct cultural exposure may reduce the relative advantage of the immersive format.

**Fig. 3:**
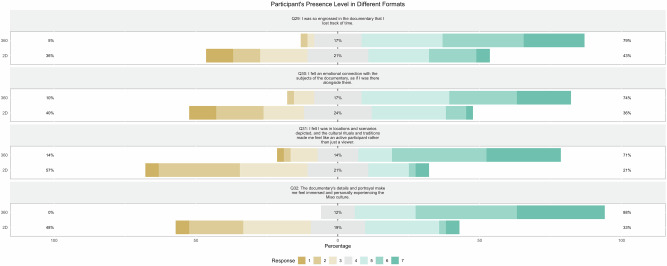
Presence levels by documentary format. This figure compares presence ratings between the 2D and 360° formats across four aspects: attention absorption (losing track of time), emotional connection, perceived presence in the environment, and overall immersion. Participants rated each aspect on a 7-point Likert scale from 1 (Strongly Disagree) to 7 (Strongly Agree). The figure was generated using R Studio.

As illustrated in Fig. 3, participants who experienced the 360° documentary consistently reported higher levels of presence compared to those who viewed the 2D format. Specifically, participants in the 360° condition more frequently lost track of time, with an average score of 5.429, compared to 4.048 for the 2D format, indicating that the immersive experience captured attention more effectively. Emotional connection to the subjects was also stronger in the 360° condition, with an average score of 5.238 versus 3.714 for the 2D format. Similarly, participants reported a greater sense of being present in the 360° environment (*M* = 5.357) compared to the 2D condition (*M* = 3.381). The depth of immersion was also enhanced, with the 360° format achieving a mean score of 5.857, significantly higher than the 2D format’s 3.690.

**Fig. 4:**
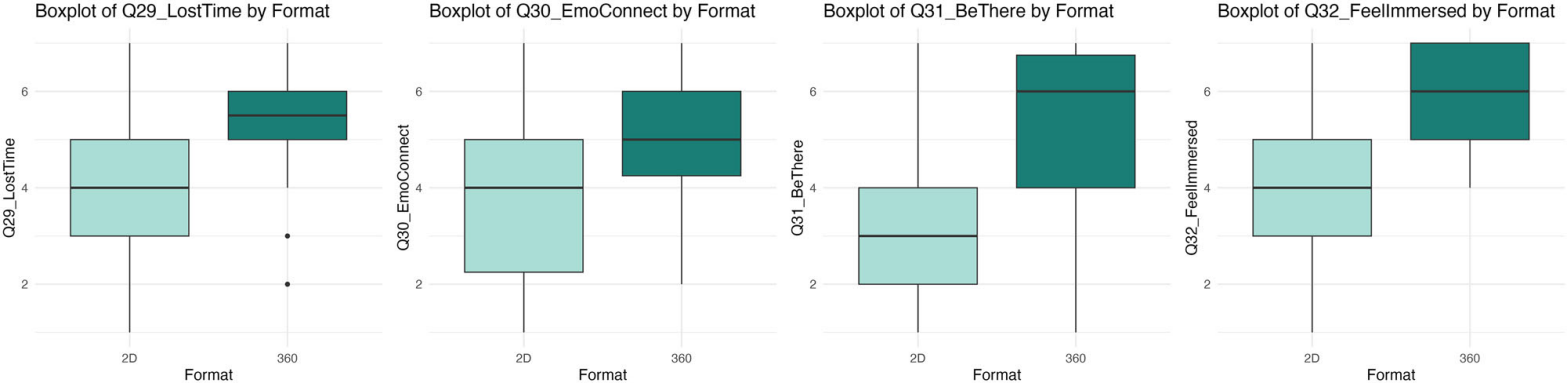
Box plots of presence measures by documentary format. This figure includes four box plots visualizing the distribution of scores for ‘Lost in Time,’ ‘Emotional Connection,’ ‘Feeling of Being There,’ and ‘Feeling of Immersion’ across the 2D and 360° formats. The plots show how the 360° format generally enhanced the sense of presence. The figure was generated using R Studio.

These patterns were supported by statistical analysis, as shown in Fig. 4. Mann-Whitney U tests confirmed significant differences between the two formats across all presence measures. Participants in the 360° condition more frequently lost track of time (*U* = 461, *p* < 0.05), felt a stronger emotional connection to the subjects (*U* = 411, *p* < 0.05), experienced a greater sense of being present in the depicted scenarios (*U* = 324, *p* < 0.05), and reported deeper immersion in Miao culture (*U* = 217, *p* < 0.05). These results demonstrate that the 360° format significantly enhanced viewer engagement across multiple dimensions of presence, from emotional resonance to the illusion of physical placement within the narrative.

Further Spearman correlation analysis indicated significant positive relationships between enjoyment and all measures of presence. Enjoyment was moderately correlated with the experience of losing track of time (r(84) = 0.400, *p* < 0.05), suggesting that higher enjoyment was associated with becoming more deeply engrossed in the documentary. Stronger correlations were observed between enjoyment and emotional connection (r(84) = 0.548, *p* < 0.05), as well as the sense of physical presence in the depicted scenarios (r(84) = 0.545, *p* < 0.05). The depth of immersion was also significantly related to enjoyment (r(84) = 0.499, *p* < 0.05). These findings indicate that participants experienced heightened enjoyment and a profound sense of presence when viewing the 360° documentary. This outcome aligns with Csikszentmihalyi’s Theory of Flow, which suggests that deep immersion and satisfaction occur when individuals are fully engaged in challenging yet attainable experiences. The strong correlations between enjoyment and presence measures suggest that the immersive documentary format effectively facilitated a flow state, enhancing both psychological engagement and satisfaction with the viewing experience.

**Fig. 5:**
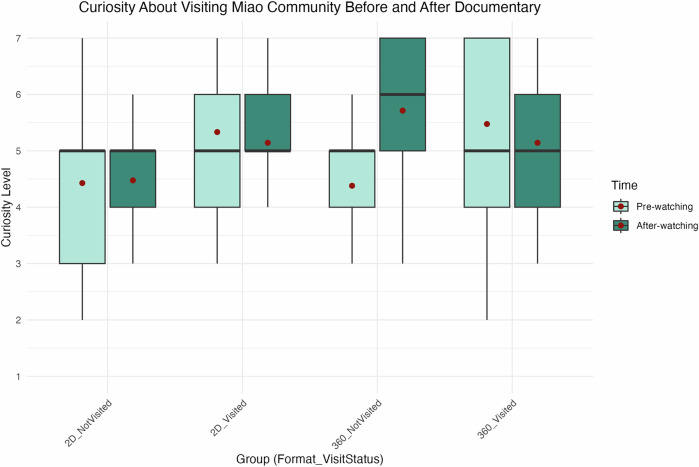
Curiosity about visiting the Miao community before and after viewing. This figure presents box plots comparing changes in participants’ curiosity about visiting the Miao community before and after watching the documentaries. The data are divided by prior visitation status and documentary format, resulting in four comparison groups. The figure was generated using R Studio.

As shown in Fig. 5, participants’ curiosity about visiting the Miao community increased after viewing the documentary, with results grouped by documentary format and prior visitation status. Participants without prior exposure demonstrated a significant increase in curiosity after watching the 360° format, as indicated by the Wilcoxon Signed-Rank Test (*p* < 0.05). This suggests that the immersive format effectively captured and sustained their interest, potentially inspiring a desire to explore Miao culture further. In contrast, participants who had previously visited Miao communities did not show a significant change in curiosity (*p* = 0.338). For the 2D format, changes in curiosity were not statistically significant for either group (visited: *p* = 0.501; not visited: *p* = 0.801), indicating that the traditional format was less effective in stimulating curiosity to the same extent.

**Fig. 6:**
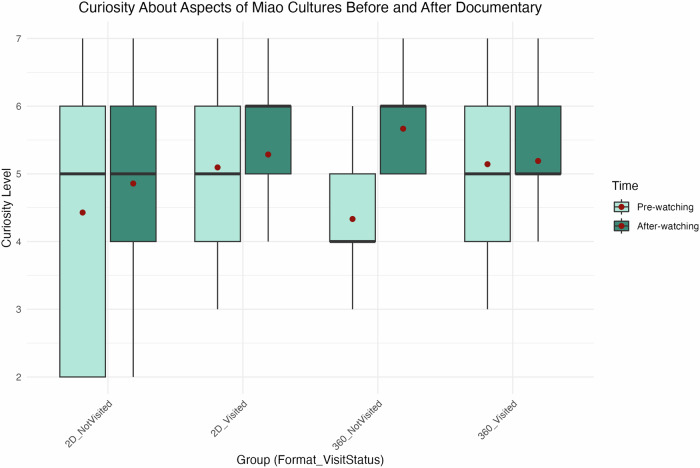
Curiosity about aspects of Miao culture before and after viewing. This figure shows box plots comparing changes in participants’ curiosity about specific aspects of Miao culture before and after viewing the documentaries. Data are grouped by prior visitation status and documentary format. The figure was generated using R Studio.

Figure 6 further illustrates changes in curiosity related to specific cultural aspects of Miao life before and after viewing the documentary. While increases in curiosity were observed across all groups, statistical significance was again found only among participants who had not visited Miao communities and experienced the 360° format (*p* < 0.05). This indicates that the immersive documentary successfully engaged these viewers and motivated them to explore specific cultural aspects more deeply. Participants who had visited Miao communities did not show a significant change (*p* = 0.790), nor did participants who viewed the 2D documentary, regardless of their visitation status (visited: *p* = 0.376; not visited: *p* = 0.360). These findings suggest that the immersive 360° format is particularly effective for audiences without prior cultural exposure, underscoring its potential value in museum contexts to promote curiosity and learning.

To further explore the factors influencing post-viewing curiosity, Spearman’s correlation analysis examined the relationships between enjoyment, presence, and curiosity outcomes. Enjoyment was moderately and significantly correlated with post-viewing interest in visiting the Miao community (r(84) = 0.517, *p* < 0.05) and in exploring cultural aspects (r(84) = 0.386, *p* < 0.05). Presence factors also demonstrated significant positive correlations with curiosity. Specifically, the experience of losing track of time was moderately correlated with both curiosity about visiting the Miao community (r(84) = 0.426) and exploring cultural aspects (r(84) = 0.391). Emotional connection showed similar correlations (r(84) = 0.412 and r(84) = 0.410), as did the sense of physically being there (r(84) = 0.453 and r(84) = 0.424), and the feeling of immersion (r(84) = 0.501 and r(84) = 0.400), all with p-values less than 0.05. These results suggest that higher enjoyment and a stronger sense of presence significantly enhance viewers’ curiosity and engagement with the cultural content presented in the documentary.

Regarding appreciation of cultural content, Mann-Whitney U tests were conducted to compare the two documentary formats. Among participants who had previously visited the Miao community, the 360° format significantly enhanced appreciation for craftsmanship compared to the 2D format (*U* = 303, *p* < 0.05). However, no statistically significant differences were observed in other appreciation measures, including the artistry and value of costumes (U = 257, *p* = 0.341), respect for practitioners (*U* = 237.5, *p* = 0.659), and the preservation of intangible cultural heritage (U = 198, p = 0.562).

**Fig. 7:**
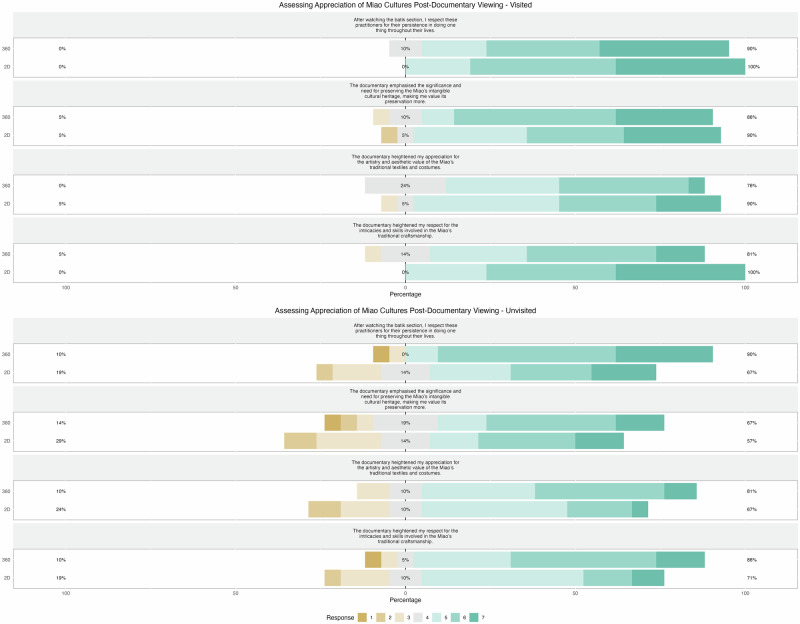
Appreciation of cultural content by prior visitation and documentary format. This figure compares participants’ appreciation of cultural content based on prior visitation and the documentary format viewed. Appreciation was measured across four aspects: the artistic and aesthetic value of Miao textiles and costumes, respect for craftsmanship, recognition of artisans’ dedication, and the importance of preserving intangible cultural heritage. Ratings were provided on a 7-point Likert scale from 1 (Strongly Disagree) to 7 (Strongly Agree). The figure was generated using R Studio.

For participants who had not visited the Miao community, the Mann-Whitney U tests did not reveal significant differences between the 2D and 360° formats across any of the appreciation measures. This included respect for craftsmanship (U = 150, *p* = 0.066), respect for practitioners (*U* = 146, *p* = 0.053), appreciation for the artistry and value of costumes (*U* = 156.5, *p* = 0.096), and the significance of preserving intangible cultural heritage (*U* = 196, *p* = 0.536). These findings, illustrated in Fig. 7, suggest that while immersive media may enrich museum experiences and deepen cultural appreciation, its impact is particularly notable in enhancing perceptions of traditional craftsmanship among those already familiar with the culture.

### Open-ended responses thematic analysis

A thematic analysis of responses to the open-ended question identified three core themes: Cultural Practices, Cultural Understanding, and Tourism and Authenticity. Subthemes within each category captured participants’ observations on traditional crafts such as batik, weaving, embroidery, and daily village life. Many participants noted the authentic portrayal of the Miao community’s environment, highlighting the emotional connection formed with an elderly textile practitioner and the detailed, step-by-step depiction of batik-making. This perceived authenticity stands in contrast to typical tourist-oriented displays, which often reduce cultural arts and performances to superficial spectacles.

The documentary also challenged common stereotypes about Miao culture by connecting specific cultural practices to broader aspects of Miao identity and tradition, emphasizing the community’s commitment to preserving its heritage. While typical tourism experiences may provide only cursory glimpses into such practices, the detailed and immersive portrayal in the documentary encouraged deeper cultural reflection and inspired participants to adopt a more engaged and respectful approach to cultural exploration (see Table 3).

**Table 3:** Open-ended question thematic analysis.

Keywords	Subsection	Summary	Responses
Cultural Practices	Traditional Crafts—Batik Making	The documentary provides a detailed view of the batik-making process.	I was most impressed by the batik-making process.
			Instead of simple narratives, this documentary engages more with the Miao group and shows more detailed processes and information, such as the whole batik-making process.
			I know better the process of making batik.
	Traditional Crafts - Weaving and Embroidery	Shows the craftsmanship involved in weaving and embroidery.	Seeing the old grandma weaving and making embroidery thread by thread and stitch by stitch, which brings me closer to their everyday life.
			Embroidery technology is spread from generation to generation rather than education.
	Art and Performance—Dance	Reflects the cultural significance of Miao dance, showing how they contribute to the cultural narrative.	Folk dance ritual has its own meaning rather than a simple dance for pleasure.
			I knew Miao ethnic groups before but didn’t know the dance.
	Art and Performance—Music	Highlights the Miao music, emphasising its narrative role in preserving cultural traditions.	The meaning of folk music is more profound than I thought.
Cultural Understanding	New Perceptions	The documentary provides new information and insights, challenging stereotypes and shifting perceptions of Miao culture.	I did not know much at all about the Miao culture before. Now I understand it.
			Everything in the video was completely new information to me.
	Identity and Continuity	Shows how cultural practices preserve and maintain Miao identity.	I was shocked that they keep the traditional lifestyle so well, like the daily outfits are special with their own culture identity.
			The presentation and performance contribute to cultural and ethnic identity construction.
Tourism and Authenticity	Documentary vs. Tourism	Compares the documentary’s portrayal to tourism experiences, highlighting its authenticity and depth.	It is good to see a specific process of certain technology, in general tourism, we often see the products, but hardly ever fully observe the process in detail.
			As a tourist, I experienced the Miao culture more commercialised. It’s more attractive in the documentary as it shows more cultural background, history, and local lifestyle.
			Although there were no significant changes as I went to Miao community before, the documentary gave me a thorough introduction of Miao culture.
	Cultural Immersion	Shows how the documentary offers an immersive view of Miao culture, sparking interest in exploring further.	It is a quite good documentary with high quality and immersive experiences.

In summary, the results indicate that immersive 360° documentaries significantly enhance viewer engagement and cultural appreciation in the context of Miao culture. Quantitative findings demonstrate increased enjoyment, presence, curiosity, and appreciation, particularly among participants without prior exposure to Miao culture. The qualitative analysis further reinforces the documentary’s capacity to deliver authentic, in-depth insights into the community’s crafts and way of life. Together, these findings highlight the transformative potential of immersive technologies to improve visitor experiences and foster greater interest in ethnic minority cultures.

## Discussion

Our controlled laboratory experiment compared two documentary formats, a traditional 2D video and an immersive 360° video, both presenting same content about Miao village life. By keeping narration and scenes constant, we directly assessed the effect of medium on viewer engagement. The 360° documentary significantly enhanced multiple dimensions of engagement, as participants reported notably higher enjoyment and a stronger sense of presence in the immersive condition. This format also evoked greater curiosity and cultural appreciation, particularly among participants without prior exposure to Miao culture. These results align with emerging research in cultural heritage, which suggests that immersive, human-centric experiences deepen engagement (Argyriou et al., 2020; Škola et al., 2020). Although 360° video technology itself is not novel, its use as an immersive documentary format for representing ethnic minority cultures remains underexplored. By focusing on the Miao community, this study provides new evidence that affordable immersive storytelling can meaningfully engage audiences with diverse cultural traditions. In China, where museums often emphasise the “unity in diversity” principle of national culture (Leung, 2023), such storytelling tools could play an important role in enriching visitors’ understanding of ethnic minority heritage.

These findings can be further understood through established theories of engagement. Enjoyment is a critical driver of media engagement, sustaining viewer interest and facilitating deeper learning (Sherry, 2004; Vorderer et al., 2004). The sensory-rich environment of the 360° documentary contributed to significantly higher enjoyment than the 2D format, which likely created a positive feedback loop. According to Flow Theory (Csikszentmihalyi, 2008), when viewers experience high enjoyment and a close match between content challenge and personal interest, they become deeply absorbed in the experience. Consistent with this theory, participants in the immersive condition reported a stronger sense of presence, suggesting that the emotional impact of enjoyment strengthened their feeling of being “there.” This finding aligns with Witmer and Singer’s (1998) research showing that higher involvement amplifies the sense of presence in virtual environments. Further, emotional engagement was confirmed to play a critical role, as Baños et al. (Baños et al., 2004) demonstrated that emotional responses significantly enhance the immersive quality of media experiences.

Curiosity and intrinsic motivation also contribute significantly to engagement in immersive environments. As Berlyne (1954) theorised, curiosity is activated in environments that invite exploration and discovery. The 360° format in our study enabled viewers to freely explore visual scenes, offering greater autonomy and fostering curiosity about Miao cultural practices and surroundings. Self-Determination Theory further supports this finding. When media experiences satisfy psychological needs for autonomy, competence, and relatedness, they increase intrinsic motivation (Ryan & Deci, 2000). In this case, the immersive environment enabled viewers to explore content at their own pace, develop a deeper understanding of unfamiliar cultural practices, and feel more connected to the people and stories presented. This finding also resonates with Cuno’s (2014) argument that encyclopedic museums stimulate global curiosity by exposing visitors to diverse cultures and histories. Integrating immersive technologies into cultural institutions may further enhance visitors’ desire to explore and deeply engage with cultural content, aligning with the mission of museums to provide dynamic and meaningful learning experiences.

Our quantitative analysis confirmed these theoretical interpretations. Participants reported higher enjoyment and stronger presence in the immersive condition, and Spearman correlation analysis revealed that those who enjoyed the documentary more also experienced a heightened sense of presence. This relationship underscores how emotional engagement can amplify cognitive and perceptual immersion. The immersive documentary also had a clear impact on curiosity, particularly among participants unfamiliar with Miao culture. Their desire to learn more about Miao traditions increased significantly after viewing the 360° documentary, while those who viewed the 2D version did not exhibit similar changes. Qualitative responses to open-ended questions reinforced this finding, as participants described how the immersive experience allowed them to notice previously unseen details and inspired further exploration. These personal reflections offer valuable insights beyond quantitative measures and address the concern that questionnaires alone may not fully capture tacit or embodied experiences.

Cultural appreciation presented a more complex pattern. Participants who had previously visited Miao communities naturally reported high appreciation regardless of the documentary format. However, among those without such exposure, the immersive format generated higher appreciation scores compared to the traditional 2D experience. This suggests that while direct cultural experiences remain the most effective means of fostering appreciation, immersive media can help bridge that gap for audiences unfamiliar with minority cultures. These findings highlight the potential of 360° documentaries to enhance understanding and appreciation of cultural heritage among broader audiences.

From a practical perspective, our findings demonstrate that immersive 360° documentaries can serve as powerful tools for making cultural heritage more accessible and engaging in museum settings. Museums interested in presenting ethnic minority cultures could integrate 360° documentaries alongside traditional displays to enrich visitor experiences. Rather than relying solely on physical exhibits, museums might offer visitors opportunities to engage with cultural narratives through immersive visual media that provide rich contextual environments. This approach allows visitors to experience cultural practices in ways that static displays cannot achieve, potentially inspiring deeper reflection and learning.

At the same time, it is important for museums to ensure that immersive technologies remain accessible to a wide range of visitors. Older audiences and those with lower digital literacy may face challenges in using head-mounted displays or interacting with unfamiliar technologies (Feng, 2024). To address these challenges, museums can offer alternative viewing options, such as high-definition 360° videos on large screens, and provide assistance through trained staff who can guide visitors in using immersive equipment. Designing user-friendly interfaces also plays a crucial role in making immersive technologies approachable and inclusive (Komianos et al., 2024). Additionally, ensuring that these technologies are accessible to visitors with disabilities requires collaborative efforts to develop more inclusive AR and VR experiences (Creed et al., 2024). Integrating these strategies into exhibition design helps museums avoid reinforcing digital divides and ensures that innovative technologies contribute to more equitable and representative cultural experiences.

Although this study provides valuable insights, it also presents several limitations. Our participant sample primarily consisted of younger, digitally proficient individuals, limiting the generalisability of the findings across different age groups and backgrounds. Future research should engage more diverse participants to assess whether the benefits of immersive media extend to older visitors and those less familiar with digital technologies. Additionally, the study relied on self-report questionnaires, which capture conscious perceptions but may overlook more subtle behavioural or embodied experiences. Our research was focused on conscious perception and reactions from being within the media formats, which include enjoyment, sense of presence, curiosity, and appreciation of cultural content. These perceptions are best captured from direct answers to our questionnaires. There may be other behavioural and physiological measures that may be latent and could be captured by physiological devices in the future. Our future research will attempt to incorporate behavioural and physiological measures for ‘ground truthing’, and these may include eye-tracking or motion analysis, to better capture subtle immersive responses.

The laboratory setting of this study further limits its ecological validity. Although this controlled environment allowed us to isolate the effects of media format, it excluded important contextual factors found in real museum environments, such as exhibit layout, social interactions, and ambient distractions from visitors, interior climate, etc. Future studies should conduct on-site evaluations to explore how immersive technologies function within actual museum contexts. Finally, our research assessed only immediate engagement outcomes, leaving questions about long-term effects unanswered. Future studies should investigate whether the enhanced engagement observed through immersive media leads to sustained interest, knowledge retention, and continued exploration of cultural content over time.

In conclusion, this research contributes new evidence that immersive 360° documentaries can significantly improve viewer engagement, stimulate curiosity, and foster appreciation of ethnic minority cultures. While the technology itself is well-established, its application to representing underrepresented cultural narratives remains a valuable area of exploration. Addressing the current limitations through expanded participant demographics, behavioural measurement approaches, and on-site museum studies will offer deeper insights into how immersive media can support more inclusive and meaningful cultural heritage experiences.

## Conclusion

This study examined the use of immersive 360° documentaries to support cultural representation and enhance viewer engagement, with a particular focus on ethnic minority cultures. By comparing a traditional 2D format with an immersive 360° documentary under controlled laboratory conditions, the results demonstrated that the 360° format enhanced enjoyment, presence, curiosity, and cultural appreciation, especially among participants without prior exposure to Miao culture. These findings suggest that immersive documentaries can create more engaging cultural experiences by offering viewers a stronger sense of connection and presence within the narratives presented.

While these results provide initial insights into the potential of 360° media for cultural engagement, they should be interpreted within the limitations of the experimental context. The controlled laboratory setting allowed for a focused comparison of media formats but did not account for the complex social, spatial, and environmental factors present in actual museum environments. Therefore, further research conducted in real-world exhibition contexts is necessary to validate these findings and explore how immersive technologies interact with broader curatorial strategies. As digital technologies continue to evolve, their thoughtful and inclusive integration into cultural heritage practices offers promising opportunities to broaden public engagement and support the meaningful representation of diverse cultures.

## Data Availability

The data generated and analysed during this study are available from the corresponding author upon reasonable request.
